# Prognostic role of serum ammonia in patients with sepsis-associated encephalopathy without hepatic failure

**DOI:** 10.3389/fpubh.2022.1016931

**Published:** 2023-01-04

**Authors:** Lina Zhao, Shaowei Hou, Risu Na, Bin Liu, Zhiwei Wang, Yun Li, Keliang Xie

**Affiliations:** ^1^Department of Critical Care Medicine, Tianjin Medical University General Hospital, Tianjin, China; ^2^School of Biomedical Engineering and Technology, Tianjin Medical University, Tianjin, China; ^3^Department of Science and Education Department, Chifeng Municipal Hospital, Chifeng Clinical Medical College of Inner Mongolia Medical University, Chifeng, China; ^4^Department of Emergency Chongqing University Central Hospital, Chongqing Emergency Medical Center, Chongqing, China; ^5^Department of Anesthesiology, Tianjin Institute of Anesthesiology, Tianjin Medical University General Hospital, Tianjin, China

**Keywords:** serum ammonia, lactate, sepsis, sepsis-associated encephalopathy (SAE), simplified acute physiology score

## Abstract

**Objectives:**

Our previous study shows that serum ammonia in sepsis patients without hepatic failure is associated with a poor prognosis. The relationship between serum ammonia level and the prognosis of sepsis-associated encephalopathy (SAE) patients without hepatic failure remains unclear. We aimed to explore the relationship between serum ammonia levels and the prognosis of patients with SAE.

**Materials and methods:**

This study is a retrospective cohort study. We collected 465 patients with SAE admitted to the intensive care unit (ICU) from Medical Information Mart for Intensive Care IV (MIMIC IV) from 2008 to 2019. Patients with SAE were divided into a survival group (369 patients) and a non-survival group (96 patients). We used the Wilcoxon signed-rank test and the multivariate logistic regression analysis to analyze the relationship between serum ammonia levels and the prognosis of patients with SAE. R software was used to analyze the dataset.

**Results:**

The primary outcome was the relationship between serum ammonia level and hospital mortality of SAE. The secondary outcomes were the relationship between serum ammonia level and hospital stays, simplified acute physiology score (SAPS II), Charlson, Glasgow coma scale (GCS), sequential organ failure assessment (SOFA), and lactate level of SAE. The mortality of patients with SAE was 20.6%. The serum ammonia level was not significantly associated with hospital mortality, longer hospital stays, higher SAPS II and Charlson scores, and lower GCS of patients with SAE. The serum ammonia level was associated with higher SOFA scores and lactate levels in patients with SAE. The SAPS II and Charlson scores were independent risk factors for death in patients with SAE.

**Conclusion:**

Serum ammonia level was associated with higher SOFA scores and lactate levels in patients with SAE. In addition, the SAPS II and Charlson scores can be used to assess the prognosis of patients with SAE. Therefore, we should closely monitor serum ammonia, SAPS II, and Charlson levels in patients with SAE.

## 1. Introduction

Sepsis-associated encephalopathy (SAE) is a common complication in patients with sepsis. It may occur in the acute phase of sepsis or after the patient survives and is discharged. SAE is manifested as changes in cognitive function and consciousness, including decreased attention, delirium, lethargy, coma, mood changes, long-term low quality of life, and dementia (1–3). The incidence of SAE is about 50% (4). The mortality risk of patients with SAE is significantly higher than that of patients with non-SAE (5). As SAE severity increases, the mortality rate is as high as 70% (6). In addition, patients with SAE have poor prognoses. Therefore, it is essential to seek potentially modifiable factors that affect the prognosis of patients with SAE.

Serum ammonia is a critical neurotoxic molecule (7). It is associated with the poor prognosis of patients with sepsis. Yazan Numan et al. found elevated ammonia levels can be a novel biomarker for sepsis (8). In a multi-center study, Jie Zhao et al. found that the area under the curve value of the ammonia level predicting the 28-day mortality was 0.813 in patients with sepsis (9). Our previous study also showed that serum ammonia levels without hepatic failure were associated with poor clinical outcomes in patients with sepsis, and the serum ammonia without hepatic failure group had higher short-term (hospital mortality: 59.8%; 30-day mortality: 47.7%) and long-term mortality (90-day mortality: 61.7%; 1-year mortality: 67.7%) (10). Amra Sakusic, Moldovan Sabov, and others found that 4.5% of patients with hyperammonemia in the ICU have a normal liver function, and 71% have encephalopathy (11). Alexandre Sanches Larangeira et al. found that serum ammonia levels of > 100 μmol/L were associated with intracranial hypertension and higher mortality (12).

The relationship between serum ammonia level and hospital mortality of SAE is unclear. We hypothesize that serum ammonia levels are associated with a poor prognosis of patients with SAE and without hepatic failure.

## 2. Materials and methods

### 2.1. Patient

This study is a retrospective cohort study. We collected patients older than 18 years and stayed in the intensive care unit (ICU) for more than 24 h. The diagnosis process for patients with SAE is as follows: (1) Patients need to meet the diagnostic criteria of Sepsis 3.0. Sepsis was diagnosed with an acute change in the total sequential organ failure assessment (SOFA) score of ≥2 and documented or suspected infection complied with the Sepsis 3.0 criteria. The patients with infection sites or prescriptions of antibiotics and samples of bodily fluids for microbiological culture had suspected infection. In line with the existing literature, the microbiological sample must have been collected within 24 h when the antibiotic was first administered, and at the first occurrence of microbiological sampling, the antibiotic administration would be within 72 h (13). (2) In patients with sepsis, we collected serum ammonia and excluded patients diagnosed with acute and chronic liver disease. (3) In patients with sepsis, traumatic brain injury, encephalitis, intracranial infection, ischemic stroke, and metabolic encephalopathy caused by severe electrolyte imbalances or glycemic disturbances, pulmonary encephalopathy caused by excessive carbon dioxide partial pressure, hepatic encephalopathy, hypertensive encephalopathy, and other liver disease or kidney disease is affecting consciousness; mental disorders and neurological disease; chronic alcohol or drug abuse were excluded by us. The diagnosis of SAE is defined according to the following three aspects: the patient's Glasgow coma scale (GCS) score of <15, patients diagnosed with delirium according to the ICD code, and patients treated with haloperidol during hospitalization (4, 14, 15).

### 2.2. Data collection

Data were retrieved FROM Medical Information Mart for Intensive Care IV (MIMIC IV) from 2008 to 2019. MIMIC-IV is a publicly available database. Applying the MIMIC IV database requires one to become a credentialed user on PhysioNet and the completion of a training course in human subjects research. In addition, we need to sign the data use agreement (DUA). The following CITI program course was completed: CITI 33690380. My registry form URL is https://physionet.org/settings/credentialing/. MIMIC IV was approved by the Institutional Review Boards of the Massachusetts Institute of Technology and Beth Israel Deaconess Medical Center. The requirement for individual patient consent was waived because the project does not impact clinical care, and all patient confidential information was anonymized. The MIMIC IV database (version 1.0) is publicly available at https://mimic-iv.mit.edu/. Any researcher who adheres to the data use requirements is permitted access to these databases. The codes are available at https://github.com/MIT-LCP/mimic-iv. We used the data of the patient's first stay in the ICU and retrieved the patient's relevant data through subject_id. The patient's age, gender, coexisting illness, site of infection, microbiology type, mechanical ventilation, renal replacement therapy, use of vasopressors, length of hospital stays, laboratory parameters, the worst laboratory parameters in the first 24 h of staying in the ICU, and the first 24-h Sequential Organ Failure Assessment (SOFA) score, Simplified Acute Physiology Score II (SAPS) score, and Glasgow Coma Scale (GCS) were extracted by R statistical software.

### 2.3. Statistical analysis

We used the Shapiro–Wilk test to evaluate whether the data were normally distributed. The continuous variables in this study were all skewed distributions. Continuous variables were expressed as the median (P 25, P 75) (interquartile range, IQR). Categorical variables were expressed as counts and proportions. We used the Wilcoxon signed-rank test to compare the continuous variables of the two groups of patients (Table 1), and the relationship between serum ammonia and GCS, SAPS II, SOFA, Charlson, lactate, and length of hospital stays (**Figures 2**, **3**). We used the Pearson exact test to compare the categorical variables of the two groups, including gender, coexisting illness (hypertension, diabetes, respiration, cardiovascular, and renal), site of infection, microbiology type, mechanical ventilation, renal replacement therapy, and use of vasopressors (Table 1). A multivariate logistic regression analysis was used to explore the risk factors of mortality in patients with SAE as shown in Table 2. The data analysis in this study was used by R software.

**Table 1:** Baseline characteristics and outcome of patient with SAE.

		**Survival group *n =* 369**	**Non-survival group *n =* 96**	**P**
**Baseline variables**				
Age, median (IQR)		64(55–72)	65(54.3–72.8)	0.351
**Gender [*****n*** **(%)]**				
	Female	145(39.3)	48(50.0)	0.058
	Male	224(60.7)	48(50.0)	
**Coexisting illness [*****n*** **(%)]**				
	Charlson	5(3-8)	6(4-9)	0.040
	Hypertension	50(13.6)	7(7.3)	0.096
	Diabetes	112(30.4)	22(2.9)	0.152
	Respiration	70(19.0)	21(21.9)	0.523
	Cardiovascular	100(27.1)	30(31.3)	0.420
	Renal	79(21.4)	17(17.7)	0.425
**Site of infection [*****n*** **(%)]**				
	Intestinal	11(3.0)	3(3.1)	0.941
	Urinary	18(4.9)	6(6.3)	0.588
	Lung	15(4.1)	4(4.2)	0.964
	Catheter	5(1.4)	2(2.1)	0.602
	Skin and soft tissue	21(5.7)	4(4.2)	0.555
	Abdomiol cavity	11(3.0)	4(4.2)	0.558
**Microbiology type [*****n*** **(%)]**				
	*Klebsiella*	45(12.2)	6(6.3)	0.097
	*Acinetobacter baumannii*	3(0.8)	1(1.1)	0.825
	*Escherichia Coli*	79(21.4)	9(9.4)	0.007
	*Pseudomonas aeruginosa*	24(6.5)	8(8.3)	0.528
	*Staphylococcus aureus*	6(1.6)	2(2.1)	0.759
	*Enterococcus*	148(40.1)	41(42.7)	0.644
**Laboratory parameters, median (IQR)**				
	Alanineamino transferase (IU/L)	40(20–41)	39.5(18.3 59.3)	0.694
	Aspartate aminotransferase (IU/L)	48(24–53.5)	48(31–59)	0.113
	Albumin(g/dL)	3.2(2.6–3.7)	1.7(0.8–3.3)	0.006
	Bilirubin(mg/dL)	1.3 (0.5– 1.7)	3.0(2.4– 3.4)	0.003
	White blood cell ( × 10^∧^^9^/L)	11.0(7.7–15.2)	11.4(7.8– 17.8)	0.329
	Neutrophils (%)	71.6(65.9–79.3)	74.6(68.9–83.0)	0.026
	Lymphocyte (%)	17.2(10.6–20.2)	14.9(6.1–20.6)	0.021
	Ammonia(μmol/L)	41(31–62)	42.5(26.3–65.8)	0.317
	Lactates (mmol/l)	1.7(1.2–2.4)	2.1(1.4–3.1)	0.002
Mechanical ventilation [*n* (%)]		186(50.4)	186(50.4)	64(66.7)
Renal replacement therapy [*n* (%)]		28(7.6)	13(13.5)	0.067
**Score system, median (IQR)**				
	SAPS II	37(29–46)	44(35.3–58.8)	<0.001
	SOFA	6(4–9)	8.5(6–12)	<0.001
	GCS	11(7–13)	8.5(4–13)	0.001
Use of vasopressors [*n* (%)]		127(34.4)	127(34.4)	55(57.3)
Length of hospital stays, days, median (IQR)		4.1(1.9–11.3)	4.1(1.9–11.3)	7.2(2.1–13.0)

**Table 2:** Multivariate logstic regression analysis of hospital mortality in SAE patients.

			**Multivariate analysis**			

		**Wald chi-square value**	***P***	**OR**	**95.0% CI**	
					**Lower**	**Upper**
Sex [*n* (%)]		4.999	0.025	1.784	1.074	2.963
Charlson		4.144	0.042	1.086	1.003	1.175
**Microbiology type [*****n*** **(%)]**						
	Klebslella	1.744	0.187	0.521	0.198	1.371
	*Escherichia coli*	6.151	0.013	0.365	0.165	0.809
**Laboratory parameters, median (IQR)**						
	Albumin(g/dL)	1.402	0.236	0.805	0.563	1.152
	Bilirubin(mg/dL)	5.077	0.024	1.142	1.017	1.282
	Neutrophils (%)	0.190	0.663	0.995	0.975	1.016
	Lymphocyte (%)	0.124	0.725	0.996	0.974	1.019
	Ammonia(μmol/L)	1.053	0.305	0.998	0.993	1.002
	Lactates (mmol/l)	0.315	0.575	1.037	0.913	1.178
Mechanical ventilation [*n* (%)]		00.107	00.744	1.108	0.599	2.048
Renal replacement therapy [*n* (%)]		0.096	0.757	0.878	0.387	1.994
Use of vasopressors [*n* (%)]		0.894	0.344	1.341	0.730	2.462
**Score system**						
	SAPS II	7.082	0.008	1.026	1.007	1.046
	SOFA	3.481	0.062	1.080	0.996	1.172
	GCS	3.578	0.059	0.938	0.878	1.002

## 3. Results

### 3.1. Baseline characteristics

**Figure 1:**
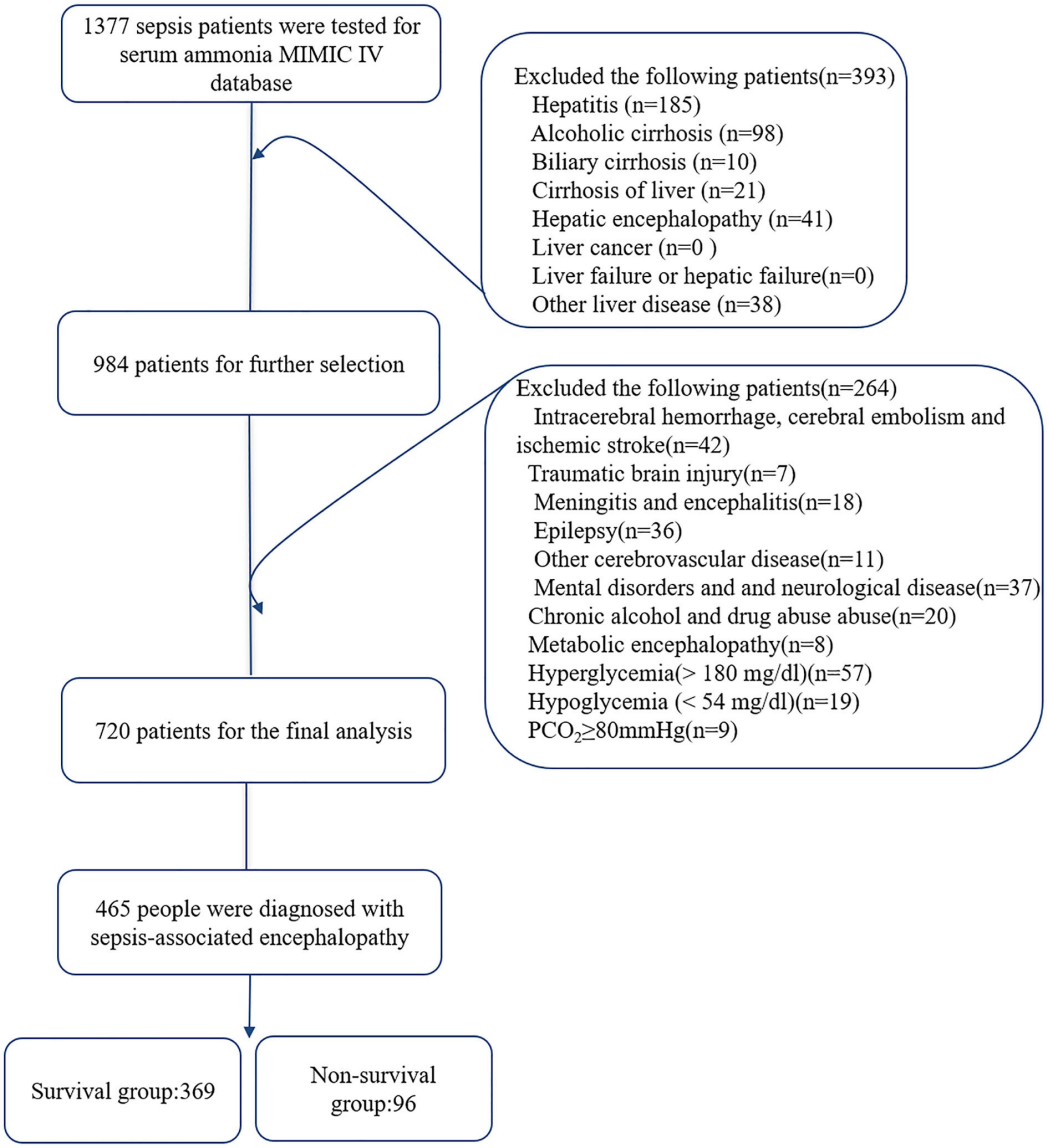
Flowchart of patient selection. MIMIC-IV, Medical Information Mart for Intensive Care IV.

Among 69,619 ICU patients, 19,658 patients met the diagnosis of sepsis 3.0. Serum ammonia was found in 1,377 of 19,658 patients with sepsis. After inclusion and exclusion criteria, 465 patients were diagnosed with SAE, divided into a survival group and a non-survival group according to hospital mortality. The survival group was 369 patients, and the non-survival group was 96 patients (Figure 1). Table 1 analyzes the baseline data and results of the survival group and non-survival group of patients with SAE. Comparing the survival group, the patients of the non-survival group had a higher Charlson score (*p* = 0.040), neutrophils [(*p* = 0.026), lactates (*p* = 0.002), SAPS II (*p* < 0.001), and SOFA (*p* < 0.001), lower GCS score (*p* < 0.001), longer length of hospital stays (*p* = 0.065), and more non-survival patients with SAE used mechanical ventilation (*P* = 0.004), renal replacement therapy (*p* = 0.067), and vasopressors (*p* < 0.001). There was no significant difference in serum ammonia levels between the two groups.

### 3.2. Multivariate regression analysis of hospital mortality in patients with SAE

Multivariate regression analysis in patients with SAE found that serum ammonia levels were not related to the prognosis of patients with SAE, and SAPS II had a better predictive value for the mortality of patients with SAE (*p* = 0.008). However, the higher the Charlson SAE patients, the worse the prognosis (*p* = 0.042) (Table 2).

### 3.3. The relationship between serum ammonia and disease severity scores of patients with SAE

**Figure 2:**
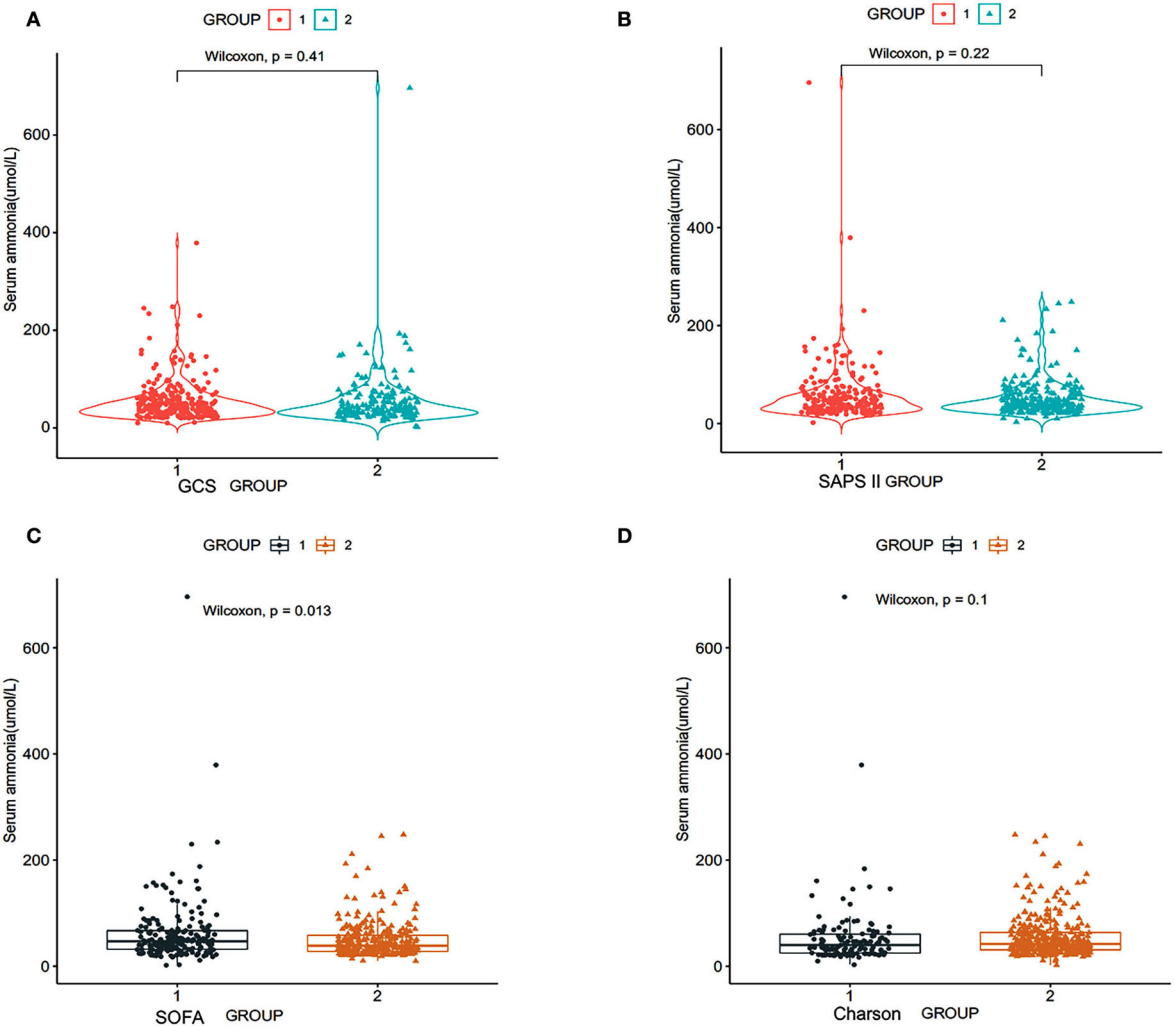
Relationship between serum ammonia and SOFA scores of patients with SAE, SAPS II, GCS, and Charlson scores. SOFA, sequential organ failure assessment; SAPS II, simplified acute physiology score; GCS, Glasgow coma scale (RStudio, Version 1.3.1056, USA). **(A)** Group 1: GCS score of patients ≥9 scores, group 2: GCS score of patients ≤8 scores; **(B)** group 1: SAPS II score of patients ≥40 scores, group 2: SAPS II score of patients ≤39 scores; **(C)** group 1: SOFA score of patients: ≥8 scores, group 2: SOFA score of patients ≤7 scores; **(D)** group 1: Charlson score of patients ≥8 scores, group 2: Charlson score of patients ≤7 scores.

According to the patient's GCS scores, the patients were divided into two groups: group 1: GCS score of patients ≥9 scores, group 2: GCS score of patients ≤8 scores. The Wilcoxon test was used to clarify the relationship between serum ammonia level and the GCS score. The study results in Figure 2 show that there is no difference in serum ammonia levels and GCS (*p* = 0.41) (Figure 2A).

We divided the patients into two groups according to the SAPS II score of the patients: group 1: SAPS II score of patients ≥40 scores and group 2: SAPS II score of patients ≤39 scores. Figure 2B shows that there is no significant correlation between the serum ammonia level and SAPS II score (*p* = 0.22) (Figure 2B).

According to the SOFA score of the patients, we divided the patients into two groups: group 1: SOFA score of patients ≥8 scores and group 2: SOFA score of patients ≤7 scores. Figure 2C shows that serum ammonia levels may be related to higher SOFA scores (*p* = 0.013).

According to the Charlson score, the patients were divided into two groups: group 1: Charlson score of patients ≥8 scores and group 2: Charlson score of patients ≤7 scores. Figure 2D shows that serum ammonia level did not correlate with the Charlson score (*p* = 0.22).

### 3.4. The relationship between serum ammonia values, lactate values of patients with SAE, and length of hospital stays

**Figure 3:**
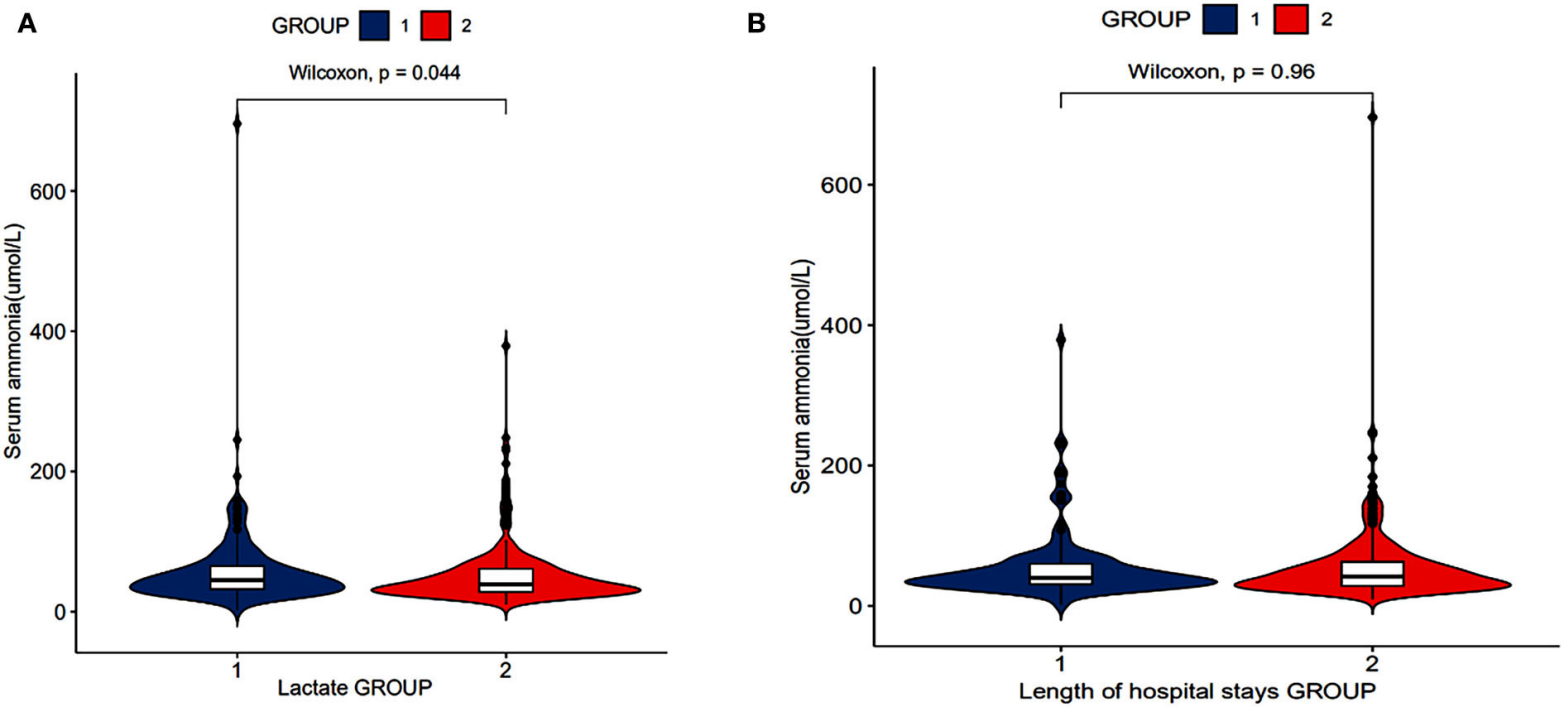
Relationship between serum ammonia and length of hospital stays, lactates of patients with SAE (RStudio, Version 1.3.1056, USA). **(A)** The relationship between serum ammonia levels and lactate levels. **(B)** The relationship between serum ammonia levels and length of hospital stays.

According to the lactate level, the patients were divided into two groups: group 1: lactates level of patients ≥2 mmol/L and group 2: lactates level of patients ≤1.9 mmol/L. Figure 3A shows that serum ammonia levels may be related to lactate levels (*P* = 0.044).

According to the length of hospital stays, the patients were divided into two groups: group 1: length of hospital stays of patients ≥10 days and group 2: length of hospital stays of patients ≤9.9 days. There was no significant difference in serum ammonia levels between the two groups (Figure 3B).

## 4. Discussion

Our cohort study shows that the non-survival group has higher Charlson, SAPS II, SOFA scores, and lactate levels; Charlson and SAPS II scores were independent risk factors for death in patients with SAE. Serum ammonia level was not associated with hospital mortality, longer hospital stays, higher SAPS II and Charlson scores, and lower GCS scores of patients with SAE without hepatic failure. However, it was associated with higher SOFA scores and lactate levels.

Our cohort study showed that the hospital mortality of non-surviving patients with SAE (20.6%) is lower than the results of Romain Sonneville et al. (50.3%) (4). It may be attributed to differences in the study population. The populations of our cohort study with acute and chronic liver disease were excluded. Although our study results show that the hospital mortality of patients with SAE is lower than in other studies, it is still at a high level. Non-survival patients with SAE had higher Charlson and SOFA scores, indicating that the more diseases in patients with SAE, the more severe organ dysfunction and the more prone to die. Non-survival patients with SAE had higher lactate levels, indicating that patients with SAE and poor perfusion were more prone to die. Zhiqiang Liu et al. found that the mortality rate of patients with sepsis in the higher lactate group was significantly higher than that of patients with sepsis in the lower lactate group (16). Our study results are consistent with their study. Yunlong Liu et al. (17) found that the sensitivity of lactate level of the 28-day mortality prediction of patients with sepsis was 0.826 (17). It shows that the worse the tissue perfusion, the more likely to die in patients with sepsis (18).

Multivariate regression analysis results show that SAPS II and Charlson scores were independent risk factors for death in patients with SAE. The relationship between SAPS II score and hospital mortality was developed using data from 137 intensive care units in 12 countries across Europe/North America (19). Amina Godinjak et al. found that SAPS II and Acute Physiology and Chronic Health Evaluation II scoring systems have the same good prognostic assessment capabilities for patients with sepsis (20). Our study shows that SAPS II had an excellent ability to assess the prognosis of patients with SAE. The Charlson scores showed a high ability to identify patients' survival (0.91) in a large healthcare database of more than 6 million hospitalized patients (21). The results of our cohort study confirm previous studies.

Our cohort study further demonstrated that higher SOFA scores and lactate levels might be related to serum ammonia levels in patients with SAE. In patients with liver failure, the patient's brain lactate levels increased significantly (22). The study by Chavarria et al. (23) found that the brain tissue and cerebrospinal fluid of rats with acute liver failure have higher levels of lactates (23). After treating brain astrocytes with NH4Cl for 24 h, the intracellular lactate level increased (22). Hyperammonemia increases the production of lactic acid in astrocytes by inhibiting the tricarboxylic acid cycle (24). Lactate causes astrocyte edema by regulating the pH value and the expression of aquaporin 4 in the brain (25). Ammonia could cause lactate levels to rise. Our study found that there is a correlation between serum ammonia and lactate levels in patients with SAE and without hepatic failure. Is there a relationship between serum ammonia in patients without hepatic failure and lactate levels in the cerebrospinal fluid of patients with SAE? Whether serum ammonia increases lactate levels needs to be confirmed by prospective studies in patients with SAE. Bodin Khwannimit et al. found that the area under the receiver operating characteristic curve of SOFA scores for predicting mortality in adults with sepsis and patients with septic shock was 0.880 (26). Jiayi Chen et al. (27) found that SOFA score was an independent risk factor for 28-day mortality in patients with SAE (27). Our study found that the SOFA scores of non-surviving patients with SAE are significantly higher than that of surviving patients with SAE (Table 1). Therefore, the SOFA scores may be an indicator for evaluating the prognosis of patients with SAE (Figure 2C). In addition, our further study found that serum ammonia levels are related to higher SOFA scores in patients with SAE. Therefore, we should closely monitor the changes in SOFA scores in patients with SAE.

There are several limitations in the study. First, the definition of SAE is based on the relevant retrospective analysis of the literature. Lack of imaging data may cause SAE's cohort to expand. Second, our study shows that the serum ammonia level is related to the SOFA score and lactates of patients with SAE. It is a retrospective study. We cannot prove its causality. Last, the condition of critically ill patients is critical and complex, and many confounding factors cannot be ruled out.

## 5. Conclusion

Non-survival patients with SAE had higher SOFA scores and lactate levels. Serum ammonia level is associated with higher SOFA scores and lactate levels in patients with SAE without hepatic failure. SAPS II and Charlson scores are valuable evaluation indicators for the poor prognosis of patients with SAE. Therefore, we should monitor the serum ammonia level, SOFA scores, SAPS II scores, and Charlson scores of patients with SAE and intervene in time.

## Data availability statement

Publicly available datasets were analyzed in this study. This data can be found at: https://mimic-iv.mit.edu.

## Code availability

Software application or custom code. The codes are available at https://github.com/MIT-LCP/mimic-iv.

## Ethics statement

The establishment of the database was approved by the Institutional Review Boards of the Massachusetts Institute of Technology and Beth Israel Deaconess Medical Center.

## Author contributions

LZ, YL, SH, and KX designed the study, analyzed it, and drafted the article. BL, ZW, and RN acquired the data. LZ wrote the first draft of the manuscript. YL and KX revised the article and worked on the English and made the final version. All authors read and approved the final manuscript.

## References

[1] Chung HY Wickel J Brunkhorst FM Geis C. Sepsis-associated encephalopathy: from delirium to dementia? J Clin Med. (2020) 9:703. 10.3390/jcm9030703 32150970 PMC7141293

[2] Molnár L Fülesdi B Németh N Molnár C. Sepsis-associated encephalopathy: a review of literature. Neurol India. (2018) 66:352–61. 10.4103/0028-3886.227299 29547154

[3] Simone C. Tauber, Marija Djukic, Johannes Gossner, Helmut Eiffert, Wolfgang B, Nau R. Sepsis-associated encephalopathy and septic encephalitis: an update. Exp. Rev. Anti-Inf. Ther. (2021) 19:215–31. 10.1080/14787210.2020.1812384 32808580

[4] Sonneville R de Montmollin E Poujade J Garrouste-Orgeas M Souweine B Darmon M. Potentially modifiable factors contributing to sepsis-associated encephalopathy. Intensive Care Med. (2017) 43:1075–84. 10.1007/s00134-017-4807-z 28466149

[5] Feng Q Ai YH Gong H Wu L Ai ML Deng SY. Characterization of sepsis and sepsis-associated encephalopathy. J Intensive Care Med. (2019) 34:938–45. 10.1177/0885066617719750 28718340

[6] Gofton TE Young GB. Sepsis-associated encephalopathy. Nat Rev Neurol. (2012) 8:557–66. 10.1038/nrneurol.2012.183 22986430

[7] Balzano T Arenas YM Dadsetan S Forteza J Felipo V. Sustained hyperammonemia induces TNF-a IN Purkinje neurons by activating the TNFR1-NF-κB pathway. J Neuroinflamm. (2020) 17:1–22. 10.1186/s12974-020-01746-z 32087723 PMC7035786

[8] Numan Y Jawaid Y Hirzallah H Kusmic D Megri M Aqtash O. Ammonia vs. lactic acid in predicting positivity of microbial culture in sepsis: the ALPS pilot study. J Clin Med. (2018) 7:182. 10.3390/jcm7080182 30049989 PMC6111562

[9] Jie Zhao Y Ping Xu Junzhao Liu Sheng Ye Cao Y. Serum ammonia levels on admission for predicting sepsis patient mortality at D28 in the emergency department: a 2-center retrospective study. Medicine. (2001) 99:e19477. 10.1097/MD.0000000000019477 32176079 PMC7220506

[10] Zhao L Gao Y Guo S Lu X Yu S Ge Z. Prognosis of patients with sepsis and non-hepatic hyperammonemia: a cohort study. Med Sci Monit. (2020) 26:e928573. 10.12659/MSM.928573 33373333 PMC7777151

[11] Sakusic A Sabov M McCambridge AJ Rabinstein AA Singh TD Mukesh K. Features of adult hyperammonemia not due to liver failure in the ICU. Crit Care Med. (2018) 46:e897–903. 10.1097/CCM.0000000000003278 29985210 PMC6095817

[12] Larangeira AS Tanita MT Dias MA Filho OFF Delfino VDA Cardoso LTQ. Analysis of cerebral blood flow and intracranial hypertension in critical patients with non-hepatic hyperammonemia. Metab Brain Dis. (2018) 33:1335–42. 10.1007/s11011-018-0245-z 29725955

[13] Liu CW Iwashyna TJ Brunkhorst FM Rea TD Scherag A Rubenfeld G. Assessment of clinical criteria for sepsis: for the third international consensus definitions for sepsis and septic shock (Sepsis-3). JAMA. (2016) 315:762–74. 10.1001/jama.2016.0288 26903335 PMC5433435

[14] Iwashyna TJ Ely EW Smith DM Langa KM. Long-term cognitive impairment and functional disability among survivors of severe sepsis. JAMA. (2010) 304:1787. 10.1001/jama.2010.1553 20978258 PMC3345288

[15] Zhao L Li Y Wang Y Gao Q Ge Z Sun X. Development and validation of a nomogram for the prediction of hospital mortality of patients with encephalopathy caused by microbial infection: a retrospective cohort study. Front Microbiol. (2021) 12:737066. 10.3389/fmicb.2021.737066 34489922 PMC8417384

[16] Liu Z Meng Z Li Y Zhao J Wu S Gou S. Prognostic accuracy of the serum lactate level, the SOFA score and the qSOFA score for mortality among adults with Sepsis. Scandinavian J Trauma Res. (2019) 27:51. 10.1186/s13049-019-0609-3 31039813 PMC6492372

[17] Liu Y Zheng J Zhang D Jing L. Neutrophil-lymphocyte ratio and plasma lactate predict 28-day mortality in patients with sepsis. J Clin Lab Anal. (2019) 33:e22942. 10.1002/jcla.22942 31265174 PMC6757133

[18] Chen W You J Chen J Zhu Y. Combining the serum lactic acid level and the lactate clearance rate into the CLIF-SOFA score for evaluating the short-term prognosis of HBV-related ACLF patients. Expert Rev Gastroenterol Hepatol. (2020) 14:1–7. 10.1080/17474124.2020.1763172 32432893

[19] Le Gall JR Lemeshow S Saulnier F A. new Simplified Acute Physiology Score (SAPS II) based on a European/North American multicenter study. JAMA. (1993) 270:2957–63. 10.1001/jama.270.24.2957 8254858

[20] Godinjak A Iglica A Rama A Tančica I Jusufović S Ajanović A. Predictive value of SAPS II and APACHE II scoring systems for patient outcome in a medical intensive care unit. Acta Med Acad. (2016) 45:97–103. 10.5644/ama2006-124.165 28000485

[21] Bannay A Chaignot C Blotière PO Basson M Weill A Ricordeau P. The best use of the charlson comorbidity index with electronic health care database to predict mortality. Med Care. (2016) 54:188–94. 10.1097/MLR.0000000000000471 26683778

[22] Tofteng F Jorgensen L Hansen BA Ott P Kondrup J Larsen FS. Cerebral microdialysis in patients with fulminant hepatic failure. Hepatology. (2010) 36:1333–40. 10.1002/hep.1840360607 12447856

[23] Bone RC Sibbald WJ Sprung CL. The ACCP-SCCM consensus conference on sepsis and organ failure. Chest. (1992) 101:1481–3. 10.1378/chest.101.6.1481 1600757

[24] Rao KV Norenberg MD. Cerebral energy metabolism in hepatic encephalopathy and hyperammonemia. Metab Brain Dis. (2001) 16:67–78. 10.1023/A:1011666612822 11726090

[25] Morishima T Aoyama M Iida Y Yamamoto N Hirate H Arima H. Lactic acid increases aquaporin 4 expression on the cell membrane of cultured rat astrocytes. Neurosci Res. (2008) 61:18–26. 10.1016/j.neures.2008.01.005 18406487

[26] Khwannimit B Bhurayanontachai R Vattanavanit V. Comparison of the accuracy of three early warning scores with SOFA score for predicting mortality in adult sepsis and septic shock patients admitted to intensive care unit. Heart Lung. (2019) 48:240–4. 10.1016/j.hrtlng.2019.02.005 30902348

[27] Chen J Shi X Diao M Jin G Zhu Y Hu W. A retrospective study of sepsis-associated encephalopathy: epidemiology, clinical features and adverse outcomes. BMC Emerg Med. (2020) 20:77. 10.1186/s12873-020-00374-3 33023479 PMC7539509

